# Application of metagenomic next-generation sequencing in pathogen detection of lung infections

**DOI:** 10.3389/fcimb.2025.1513603

**Published:** 2025-05-01

**Authors:** Song Chen, Tanglin Ouyang, Kaiyang Wang, Xuan Hou, Rong Zhang, Meiyong Li, Haibin Zhang, Qinghua He, Xiuzhen Li, Zezhang Liu, Xiaozhong Wang, Bo Huang

**Affiliations:** ^1^ Jiangxi Province Key Laboratory of Immunology and Inflammation, Jiangxi Provincial Clinical Research Center for Laboratory Medicine, Department of Clinical Laboratory, The Second Affiliated Hospital, Jiangxi Medical College, Nanchang University, Nanchang, Jiangxi, China; ^2^ School of Public Health, Nanchang University, Nanchang, Jiangxi, China; ^3^ Department of Emergency, The Second Affiliated Hospital, Jiangxi Medical College, Nanchang University, Nanchang, Jiangxi, China; ^4^ BGI Genomics, Shenzhen, Guangzhou, China; ^5^ Clinical Laboratory, BGI Genomics, Wuhan, Hubei, China

**Keywords:** metagenomic next-generation sequencing, lung infection, bronchoalveolar lavage fluid, conventional microbiological test, pathogens diagnosis, antibiotic treatment adjustment

## Abstract

**Background:**

Metagenomic next-generation sequencing (mNGS) has been widely reported to provide crucial information for the diagnosis and treatment of infectious diseases. In this study, we aimed to evaluate mNGS in pathogens diagnosis of lung infections.

**Methods:**

A total of 188 patients who were suspected of pulmonary infection and received medical treatment at the Second Affiliated Hospital of Nanchang University from August 2022 to December 2023 were enrolled in this study. Conventional microbiological tests (CMTs) and mNGS were employed for pathogens diagnosis.

**Results:**

Statistical results indicated that mNGS were significantly better than CMTs in sensitivity, negative predictive value, and negative likelihood ratio. Remarkably, the positive detection rate of mNGS was significantly higher than that of CMTs (86.17% *vs* 67.55%, *P* < 0.01). Through mNGS, we identified 96 pathogens, comprising 59 bacteria, 18 fungi, 15 viruses, and 4 special pathogens. In contrast, CMTs detected 28 species, including 25 bacteria and 3 fungi. The effectiveness rate of antibiotic treatment decisions based on mNGS results was 40.60%. Out of 54 cases with positive treatment impacts, mNGS results contributed to the treatment and improved prognosis of 16 infections caused by atypical pathogens.

**Conclusion:**

Our results proved the essential role of mNGS in lung infection diagnosis, enabling early detection and the prompt development of targeted anti-infection therapies. We recommended that the clinical application of mNGS can enhance treatment effectiveness and improve patient prognosis.

## 1 Introduction

Infectious diseases, caused by pathogenic microorganisms, typically present with symptoms like fever and an increased pulse rate (Prabhu, 2023). A severe infection can result in sepsis, septic shock, and even death (Delogu et al., 2011). Infectious diseases can be categorized according to the site of infection such as pulmonary infections, central nervous system infections, bloodstream infections, urinary tract infections, and gastrointestinal infections (Van Seventer and Hochberg, 2017). Among these, pulmonary infections are particularly prevalent and can result in multiple complications (Li et al., 2019). Patients with underlying conditions and compromised immune function tend to have a poorer prognosis, with pulmonary infections being one of the top ten causes of death globally (Azoulay et al., 2020). Pulmonary infections arise from various pathogens, including bacteria, viruses, fungi, and parasites (Zhao et al., 2023). Differences in environmental factors, affected populations, and disease severity contribute to the diversity of pathogens between patients. The emergence of multidrug-resistant organisms and new pathogens presents challenges in clinical diagnosis and treatment (Magill et al., 2014; Katz and Williams, 2018).

Currently, the conventional microbiological tests (CMTs) for lung pathogens detection encompass traditional microbial cultivation, antigen-antibody testing, and nucleic acid detection through polymerase chain reaction techniques. However, these CMTs often exhibit a low sensitivity in pathogen detection due to drawbacks such as long turnaround times and low detection rates, making it challenging for clinicians to determine an accurate treatment plan (Diao et al., 2022; Wu et al., 2022). Therefore, rapid and precise identification of pathogens is crucial. Metagenomic next-generation sequencing (mNGS) is a method for pathogen identification (Pham et al., 2023), offering high sensitivity, high specificity, and broad pathogen coverage, which provides critical information for pulmonary infections. Despite limitations such as lower sensitivity compared to NAATs, restricted applicability in resource-limited settings, and non-first-line status in current guidelines, this method enables rapid and precise identification of pathogens for accurate treatment, improving patient prognosis and demonstrating significant potential in clinical applications (Stratton and Tang, 2020; Liu et al., 2022; Liu, 2024).

In this study, the types of specimens used for pathogen testing of pulmonary infections included bronchoalveolar lavage fluid, blood, sputum, etc. We evaluated the diagnostic performance in pulmonary infections of mNGS, compared to CMTs. By assessing sensitivity and specificity, we determined that mNGS outperformed CMTs in diagnostic performance. Meanwhile, we detected a total of 96 pathogens through mNGS, exceeding the 28 identified by conventional culture methods. Consequently, the adjustments in antibiotic treatment decisions based on mNGS results additionally improved the treatment and prognosis of 16 patients with atypical pathogens. Our findings proved that mNGS is a powerful tool for early pathogens diagnosis in pulmonary infections, enabling timely and targeted formulation of anti-infection therapies, which in turn improves effectiveness treatment effectiveness and patient outcome.

## 2 Methods

### 2.1 Subjects of the study

This study included 188 patients with suspected pulmonary infections treated at the Second Affiliated Hospital of Nanchang University between August 2022 and December 2023. All samples were collected after obtaining informed consent from the patients. Meanwhile, this study received approval from the Second Affiliated Hospital Ethics Committee of Nanchang University. Inclusion criteria: (1) Onset in the community; (2) Presence of at least one clinical symptom associated with pulmonary infection, such as cough, sputum, hemoptysis, fever, chest pain, chest tightness, rapid breathing, difficulty breathing, wet rales, or abnormal peripheral WBC count (> 10×10^9^/L or < 4×10^9^/L); (3) Chest imaging indicating pulmonary lesions; (4) Signed informed consent for BALF mNGS testing and agreed to participate in this study; (5) Underwent both CMTs and BALF mNGS test; (6) Complete clinical data records; (7) Hospitalized patients. Experienced doctors diagnosed pulmonary infections by referring to the Chinese guidelines for adult community-acquired pneumonia (2016 edition) and evaluating laboratory and imaging results in conjunction with underlying diseases and clinical manifestations (Cao et al., 2018). Exclusion criteria: (1) Patients younger than 18 years old; (2) Patients with autoimmune diseases, other immunodeficiencies, or those receiving cancer treatment; (3) Patients with conditions such as pulmonary tuberculosis, lung tumors, interstitial lung disease, pulmonary edema, atelectasis, pulmonary embolism, pulmonary eosinophilic infiltration, and pulmonary vasculitis (Cao et al., 2018).

### 2.2 Sample collection and test

Qualified blood, sputum, and BALF samples were collected by experienced doctors following strict aseptic protocols. After preprocessing, these samples were utilized for CMTs and mNGS testing (BGI China).

CMTs included bacterial, fungal, and tuberculosis smear tests (microscopy); Pneumocystis tests (DFA/PCR); bacterial and fungal cultures; G and GM tests (immunoassays); tuberculosis antibody tests (serology); Mycoplasma pneumoniae and Chlamydia IgM antibody detection (serology); rapid screening for respiratory pathogens (multiplex PCR); screening for Influenza A and B antigens (immunochromatography); Mycobacterium tuberculosis gene detection (PCR-based); Mycobacterium tuberculosis nucleic acid tests (NAATs); and dual viral tests for human cytomegalovirus and EB virus (PCR-based).

BALF is directly obtained from the lungs, providing clear and accurate pathological information about the lungs. It slightly affected the mNGS results due to lower contamination from microorganisms colonizing the mouth and upper respiratory tract and relatively fewer human sequences (Zheng et al., 2021; Diao et al., 2022).

BGI Genomics completed the mNGS sequencing and bioinformatics analysis (BGI China). The specific methods were adapted from previously reported studies (Shen et al., 2023). Briefly, DNA or RNA samples were extracted from BALF and used for library construction. The RNA was further subjected to reverse transcription into cDNA. Following extraction, sequencing libraries were constructed by fragmentation, end repair, adapter ligation, and PCR amplification. Next, sequencing was performed on MGISEQ-2000 (BGI China) to generate raw reads. Bioinformatics analysis of sequenced data included quality control, filtering out low-quality sequences (Trimmomatic version 0.39), excluding hg19 human genome sequences (BWA version 0.7.15-r1140), aligning remaining sequences with microbial genome databases, and identifying microbes.

### 2.3 Clinical records

The demographics, underlying diseases, clinical presentations, results from CMTs and mNGS, imaging findings, initial antimicrobial treatment plans, and adjustments based on mNGS results were recorded for each patient. The clinical diagnosis was determined by infectious disease physicians according to the guidelines

### 2.4 Criteria for positive mNGS results

Specifically mapped read number (SMRN) were used to identify pathogens excluding contaminating organisms from the respiratory tract, skin, environment, and reagents. SMRN ≥ 3 was considered positive for bacteria, fungi, and viruses. SMRN ≥ 100 indicated positivity for parasites. SMRN ≥ 1 indicated positivity for Mycobacteria, Chlamydia, Mycoplasma, and Rickettsia.

### 2.5 Statistical analysis

Data were processed and analyzed using SPSS 26.0. Normally distributed continuous variables were described using mean ± standard deviation (X ± s), while non-normally distributed variables were described using the interquartile range. We evaluated the diagnostic performance of CMTs and mNGS using sensitivity, specificity, positive predictive value, negative predictive value (NPV), positive likelihood ratio, negative likelihood ratio (NLR), and receiver operating characteristic (ROC) curves and AUC. Statistical significance was defined as *P* < 0.05.

## 3 Results

### 3.1 Clinical characteristics

As shown in **Table 1**, 188 patients were included in this study, with 126 males (67.02%) and 62 females (32.98%). The mean age was 57.56 ± 16.65 years, ranging from 19 to 89 years. The primary clinical symptoms among patients were cough (141 cases, 75.00%), sputum production (110 cases, 58.51%), and fever (104 cases, 55.32%). The primary underlying conditions in patients included liver disease (84 cases, 44.68%), hypertension (59 cases, 31.38%), and diabetes (49 cases, 26.06%). Of these, 176 were diagnosed with pulmonary infection, while 12 were non-pulmonary infection patients.

**Table 1:** Clinical characteristics.

		Cases number (%)
Sex	Male	126 (67.02)
	Female	62 (32.98)
Clinical symptoms	Cough	141 (75.00)
	Sputum production	110 (58.51)
	Fever	104 (55.32)
	Chest tightness	50 (26.60%)
	Shortness of breath	31 (16.49%)
	Rapid breathing	24 (12.77%)
	Hemoptysis	19 (10.11%)
	Chest pain	11 (5.85%)
Underlying disease	Liver disease	84(44.68%)
	Hypertension	59(31.38%)
	Diabetes	49(26.06%)
Diagnosis	Pulmonary infection	176(93.62%)
	Nonpulmonary infection	12(6.38%)

### 3.2 Analysis of diagnostic performance

Diagnostic performance analysis indicated that mNGS testing outperformed CMTs in sensitivity, specificity, positive predictive value, NPV, positive likelihood ratio, and NLR (**Table 2**). Importantly, the sensitivity, NPV, and NLR of mNGS were significantly higher than those of CMTs (*P* < 0.01).

**Table 2:** Comparison of diagnostic performance.

	Sensitivity	Specificity	PPV	NPV	PLR	NLR	True positive	True negative	False positive	False negative
CMTs	68.75%(61.34%-75.51%)	50.00%(21.09%-78.91%)	95.28%(91.91%-97.28%)	9.84%(5.61%-16.68%)	1.38(0.77-2.44)	0.63(0.34-1.15)	121	6	6	55
mNGS	89.77%(84.32%-93.83%)	66.67%(34.88%-90.08%)	97.53%(94.66%-98.88%)	30.77%(19.72%-44.57%)	2.69(1.21-6.00)	0.15(0.09-0.28)	158	8	4	18
*P*	< 0.01	0.32	0.13	< 0.01	0.10	<0.01				

PPV, positive predictive value; NPV, negative predictive value; PLR, positive likelihood ratio; NLR, negative likelihood ratio.

### 3.3 Spectrum of detected pathogens

**Figure 1:**
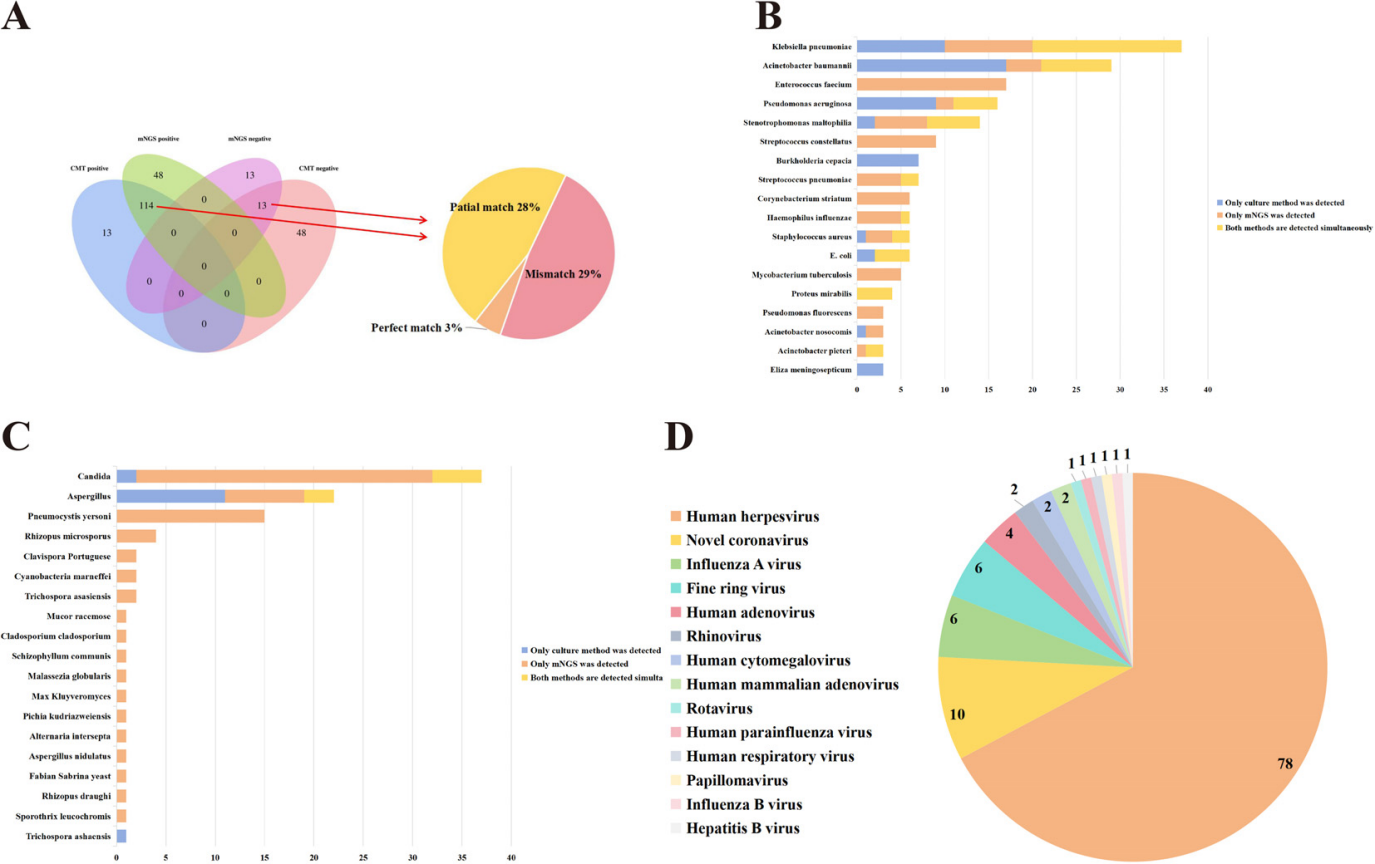
Comparison of CMTs and mNGS results. **(A)** The detection results of CMTs and mNGS. **(B)** Comparison of Culturing and mNGS results (Bacteria). **(C)** Comparison of Culturing and mNGS results (Fungi). **(D)** Virus classification identified via mNGS.

The positive detection rates for CMTs and mNGS testing were 67.55% and 86.17%, respectively, with a significant statistical difference (*P* < 0.01). Among the 188 cases, 13 were negative in both methods, 114 were positive in both methods, 48 were positive only in mNGS, and 13 cases were positive only in CMTs (**Figure 1A**). Of the 114 positive cases in both methods, 6 were completely matched, 53 were partially matched (at least one common pathogen), and 55 were completely mismatched (**Figure 1A**).

A total of 96 microorganisms were detected by mNGS, including 59 bacteria, 18 fungi, 14 viruses, and 4 special pathogens. In contrast, routine culture methods detected only 28 microorganisms, comprising 25 bacteria and 3 fungi. Specifically, mNGS showed superior detection capabilities over culture methods for bacteria such as *Enterococcus faecium, Stenotrophomonas maltophilia, Streptococcus constellatus, Streptococcus pneumoniae, Corynebacterium striatum, Haemophilus influenzae, Staphylococcus aureus, Mycobacterium tuberculosis, Pseudomonas fluorescens, Acinetobacter nosocomis*, and *Acinetobacter pieteri* (**Figure 1B**). Moreover, mNGS also showed superior detection capabilities than CMTs for fungi such as *Pneumocystis yersonl, Rhizopus microsporus* (**Figure 1C**). Compared to virus-related CMTs, mNGS identified 14 types of viruses in this study that could not be detected by CMTs, including *Human herpesvirus, Novel coronavirus* (**Figure 1D**). Hence, these results suggested that mNGS exhibited advantages in detecting fungi, viruses, and special pathogens, offering more comprehensive information.

### 3.4 Impact of mNGS on clinical decisions

Based on the mNGS results, treatment decisions included initiating or intensifying antibiotic therapy, adjusting antibiotic regimens, and reducing antibiotic use. After excluding 55 patients whose treatment decisions could not be assessed due to incomplete information, lack of patient cooperation with treatment adjustments, critical conditions, early discharge before mNGS results were available, or early discharge due to no improvement after treatment adjustment based on mNGS results, the impact of mNGS results on treatment decisions was analyzed for 133 out of 188 patients. The impact of these treatment decisions was categorized as positive impact (54/133), negative impact (1/133), and no impact (78/133). Among the 133 patients, mNGS results had a positive influence on the treatment decisions of 54 patients (40.60%), a negative impact on one patient due to unnecessary antibiotic treatment, and no impact on 78 patients (who continued with their original treatment decisions) (**Table 3**).

**Table 3:** Treatment decisions and outcomes based on mNGS detection results.

Clinical efficacy	Treatment decisions	Number
Positive impact	Antibiotic treatment or intensified antibiotic therapy	26
	Adjusting antibiotic regimens or reducing antibiotic therapy	28
Negative impact	Antibiotic treatment or intensified antibiotic therapy	1
	Adjusting antibiotic regimens or reducing antibiotic therapy	0
No impact	The mNGS results were discordant with clinical findings or had no guiding significance, so the original treatment plan was continued	19
	The mNGS results were concordant with the original treatment plan, so the original treatment plan was continued	16
	The patient’s condition had improved before the results were available, so the original treatment plan was continued	43

Among the 54 cases where mNGS results had a positive impact, the corresponding culture results were as follows: negative in 19 cases, *Klebsiella pneumoniae* in 9 cases, *Acinetobacter baumannii* and *Aspergillus* in 6 cases each, *Candida* in 5 cases, *Proteus mirabilis* in 4 cases, *Staphylococcus aureus* and *Pseudomonas aeruginosa* in 2 cases each, and *Streptococcus pneumoniae*, *Stenotrophomonas maltophilia, Moraxella catarrata, Elizabethia pyogenes meninges, Enterobacter cloacae, Acinetobacter pieteri, Providencia rei* in 1 case each. After comparing culture and mNGS results, we found that 4 cases matched, 23 cases partially matched, 26 cases did not match, and 1 case could not be assessed due to the absence of culture results. Moreover, mNGS results improved the treatment and prognosis of 16 cases infected with atypical pathogens including *Streptococcus suis, Pneumocystis jirovecii, Cyanobacteria marneffei, Mycobacterium tuberculosis, intracellular branching filamentous bacteria, Mycoplasma pneumoniae*, and *Chlamydia psittaci*. This finding further highlighted the critical role of mNGS in the adjunctive diagnosis and treatment of atypical pathogen infections.

## 4 Discussion

In recent years, mNGS has been widely applied and acknowledged in clinical pathogen detection for infectious diseases (Li et al., 2021). Due to its ability to accurately identify pathogens, mNGS has become a crucial diagnostic tool in the management of clinical infections. Simplifying detection processes and enhancing pathogen detection sensitivity, mNGS offers advantages in diagnosing infectious diseases of unknown etiology (Liu et al., 2022; Pham et al., 2023). Consequently, routine culture methods are more time-consuming, compared to mNGS (Austin, 2017; Miao et al., 2018; Simner et al., 2018; Li et al., 2019; Mu et al., 2021). Our results showed the sensitivity and specificity of CMTs were 68.75% and 50%, respectively, which were higher than previous research (Jin et al., 2022). This difference may be attributed to different CMTs and patient enrollment criteria. It has been reported that mNGS had higher detection rates for most pathogens (Chen et al., 2021; Qian et al., 2021; Wu et al., 2022; Xiao et al., 2023; Xiao et al., 2024), aligning with our findings. This study showed that the detection sensitivity and pathogen detection rate of mNGS were superior to CMTs (**Table 2**). The majority of studies showed that the specificity of mNGS is lower than CMTs (Wang et al., 2019; Huang et al., 2020; Xie et al., 2021; Wei et al., 2022). In contrast, the specificity of mNGS in this study was modestly elevated compared to CMTs, consistent with the results observed by Cai et al (Cai et al., 2020). This difference may be attributed to different types of samples used for mNGS among studies. BALF in this study is an ideal sample type for mNGS due to its minimal contamination from oral and upper respiratory tract microbes (Zheng et al., 2021; Diao et al., 2022). Additionally, the NLR of mNGS was significantly lower than that of CMTs (*P* < 0.01), aligning with previous research (Parize et al., 2017). We found 18 false-negative mNGS results, likely due to low sequence counts and low abundance, or BALF degradation during storage and transport. As for the 4 false-positive results, they may be attributed to mNGS misreporting colonizing or contaminating bacteria, or the patient recovering from a past infection without treatment (Zheng et al., 2021; Xiao et al., 2023). Moreover, our results demonstrated that mNGS detects a broader spectrum of pathogens, especially fungi and viruses, which is consistent with the conclusions of Miao et al (Miao et al., 2018), but differs from the findings of Xie et al., who reported better bacterial detection (Xie et al., 2019).

Overuse of anti-infective treatment can lead to increased pathogen resistance, decreased patient immunity, and a higher risk of secondary infections, thereby increasing the economic burden and wasting medical resources. In a multi-center prospective study of 159 cases, mNGS identified pathogenic factors in 45 cases and facilitated antibiotic de-escalation in 19 cases (Zhou et al., 2021). Similarly, in another multi-center study, mNGS significantly enhanced the diagnostic efficiency for suspected pulmonary infections (Jin et al., 2022). Notably, mNGS enabled modifications in the treatment decisions for a total of 127 patients with pulmonary infections in three studies (Mu et al., 2021; Zhou et al., 2021; Guo et al., 2022). In this study, 40.60% (54/133) of patients who adjusted their antibiotic treatment based on mNGS results experienced positive outcomes, indicating the feasibility of mNGS in assisting the clinical treatment of pulmonary infections.

Pulmonary infections caused by atypical pathogens are increasingly prevalent, often posing diagnostic challenges due to their atypical nature, lack of specific symptoms, and limitations in CMTs. Our results demonstrated that mNGS can identify atypical pathogens, including 16 cases infected with atypical pathogens such as *Mycobacterium tuberculosis, Mycobacterium intracellularis, Mycoplasma pneumoniae, Chlamydia psittaci, Pneumocystis yersoni, Cyanobacteria marneffei* and *Streptococcus suis (* Huang et al., 2021; Huang et al., 2021). These findings suggested that mNGS is a valuable tool for improving the diagnosis and management of pulmonary infections caused by atypical pathogens.

This study also has several limitations. It is retrospective with a small sample size, which may introduce bias into the results. Additionally, some patients had received antimicrobial treatment before laboratory tests, which could affect the results of CMTs and mNGS, especially those relying on culture methods. Considering the inherent limitations of mNGS and CMTs, we recommended integrating clinical characteristics, results of CMTs, pulmonary imaging findings, and other relevant factors in clinical practice to conduct comprehensive analyses and better leverage the diagnostic value of mNGS.

## 5 Conclusion

Our study results highlighted that mNGS exhibits remarkable advantages in pulmonary infection pathogens diagnosis compared to CMTs. It demonstrated higher efficiency and broader coverage, especially for atypical and rare pathogens. This technology holds crucial application value in pulmonary infection pathogen diagnosis, providing a scientific basis for clinicians to develop targeted antimicrobial strategies, thereby enhancing treatment effectiveness and improving patient outcomes. We recommended the widespread clinical application of mNGS to deliver higher-quality, personalized medical care to patients.

## Data Availability

The datasets presented in this study are available in an online repository. The names of the repository/repositories and accession number(s) can be found below: https://www.ncbi.nlm.nih.gov, PRJNA1246259.

## References

[B1] Austin B. (2017). The value of cultures to modern microbiology. Antonie van Leeuwenhoek 110, 1247–1256. doi: 10.1007/s10482-017-0840-8 28168566

[B2] Azoulay E. Russell L. Van De Louw A. Metaxa V. Bauer P. Povoa P.. (2020). Diagnosis of severe respiratory infections in immunocompromised patients. Intensive Care Med. 46, 298–314. doi: 10.1007/s00134-019-05906-5 32034433 PMC7080052

[B3] Cai Y. Fang X. Chen Y. Huang Z. Zhang C. Li W.. (2020). Metagenomic next generation sequencing improves diagnosis of prosthetic joint infection by detecting the presence of bacteria in periprosthetic tissues. Int. J. Infect. Dis. 96, 573–578. doi: 10.1016/j.ijid.2020.05.125 32505872

[B4] Cao B. Huang Y. She D. Y. Cheng Q. J. Fan H. Tian X. L.. (2018). Diagnosis and treatment of community-acquired pneumonia in adults: 2016 clinical practice guidelines by the Chinese Thoracic Society, Chinese Medical Association. Clin. Respir. J. 12, 1320–1360. doi: 10.1111/crj.2018.12.issue-4 28756639 PMC7162259

[B5] Chen Y. Feng W. Ye K. Guo L. Xia H. Guan Y.. (2021). Application of metagenomic next-generation sequencing in the diagnosis of pulmonary infectious pathogens from bronchoalveolar lavage samples. Front. Cell. Infection Microbiol. 11. doi: 10.3389/fcimb.2021.541092 PMC7991794 33777827

[B6] Delogu L. G. Deidda S. Delitala G. Manetti R. (2011). Infectious diseases and autoimmunity. J. Infect. Dev. Ctries 5, 679–687. doi: 10.3855/jidc.2061 21997935

[B7] Diao Z. Han D. Zhang R. Li J. (2022). Metagenomics next-generation sequencing tests take the stage in the diagnosis of lower respiratory tract infections. J. Advanced Res. 38, 201–212. doi: 10.1016/j.jare.2021.09.012 PMC9091713 35572406

[B8] Guo W. Cui X. Wang Q. Wei Y. Guo Y. Zhang T.. (2022). Clinical evaluation of metagenomic next-generation sequencing for detecting pathogens in bronchoalveolar lavage fluid collected from children with community-acquired pneumonia. Front. Med. 9. doi: 10.3389/fmed.2022.952636 PMC9334703 35911412

[B9] Huang H. Deng J. Qin C. Zhou J. Duan M. (2021). Disseminated Coinfection by Mycobacterium fortuitum and Talaromyces marneffei in a Non-HIV Case. Infect. Drug Resist. 14, 3619–3625. doi: 10.2147/IDR.S316881 34526784 PMC8435476

[B10] Huang J. Jiang E. Yang D. Wei J. Zhao M. Feng J.. (2020). Metagenomic next-generation sequencing versus traditional pathogen detection in the diagnosis of peripheral pulmonary infectious lesions. Infect. Drug Resist. 13, 567–576. doi: 10.2147/idr.S235182 32110067 PMC7036976

[B11] Huang T. Chen Y. Zhang J. He R. Qu D. Ye Q.. (2021). Rapid and accurate diagnosis of brain abscess caused by Nocardia asiatica with a combination of Ziehl-Neelsen staining and metagenomics next-generation sequencing. Eur. J. Neurol. 28, 355–357. doi: 10.1111/ene.14533 32920981

[B12] Jin X. Li J. Shao M. Ji N. Zhu Y. Huang M.. (2022). Improving suspected pulmonary infection diagnosis by bronchoalveolar lavage fluid metagenomic next-generation sequencing: a multicenter retrospective study. Microbiol. Spectr. 10, e0247321. doi: 10.1128/spectrum.02473-21 35943274 PMC9431624

[B13] Katz S. E. Williams D. J. (2018). Pediatric community-acquired pneumonia in the United States: changing epidemiology, diagnostic and therapeutic challenges, and areas for future research. Infect. Dis. Clin. North Am. 32, 47–63. doi: 10.1016/j.idc.2017.11.002 29269189 PMC5801082

[B14] Li N. Cai Q. Miao Q. Song Z. Fang Y. Hu B. (2021). High-throughput metagenomics for identification of pathogens in the clinical settings. Small Methods 5. doi: 10.1002/smtd.202000792 PMC7883231 33614906

[B15] Li Z. Lu G. Meng G. (2019). Pathogenic fungal infection in the lung. Front. Immunol. 10. doi: 10.3389/fimmu.2019.01524 PMC6616198 31333658

[B16] Li G. Sun J. Pan S. Li W. Zhang S. Wang Y. (2019). Comparison of the performance of three blood culture systems in a chinese tertiary-care hospital. Front. Cell. Infection Microbiol. 9, 285. doi: 10.3389/fcimb.2019.00285 PMC6698792 31456951

[B17] Liu B. M. (2024). Epidemiological and clinical overview of the 2024 Oropouche virus disease outbreaks, an emerging/re-emerging neurotropic arboviral disease and global public health threat. J. Med. Virol. 96, e29897. doi: 10.1002/jmv.29897 39221481 PMC12520762

[B18] Liu B. Tian Q. Wang P. Xu S. F. Tian Y. L. Zhao J.. (2022). Evaluating the diagnostic value of using metagenomic next-generation sequencing on bronchoalveolar lavage fluid and tissue in infectious pathogens located in the peripheral lung field. Ann. Palliative Med. 11, 1725–1735. doi: 10.21037/apm-21-3474 35672890

[B19] Magill S. S. Edwards J. R. Bamberg W. Beldavs Z. G. Dumyati G. Kainer M. A.. (2014). Multistate point-prevalence survey of health care–associated infections. New Engl. J. Med. 370, 1198–1208. doi: 10.1056/NEJMoa1306801 24670166 PMC4648343

[B20] Miao Q. Ma Y. Wang Q. Pan J. Zhang Y. Jin W.. (2018). Microbiological diagnostic performance of metagenomic next-generation sequencing when applied to clinical practice. Clin. Infect. Dis. 67, S231–S240. doi: 10.1093/cid/ciy693 30423048

[B21] Mu S. Hu L. Zhang Y. Liu Y. Cui X. Zou X.. (2021). Prospective evaluation of a rapid clinical metagenomics test for bacterial pneumonia. Front. Cell. Infect. Microbiol. 11, 684965. doi: 10.3389/fcimb.2021.684965 34737971 PMC8560692

[B22] Parize P. Muth E. Richaud C. Gratigny M. Pilmis B. Lamamy A.. (2017). Untargeted next-generation sequencing-based first-line diagnosis of infection in immunocompromised adults: a multicentre, blinded, prospective study. Clin. Microbiol. Infect. 23, 571–574. doi: 10.1016/j.cmi.2017.02.006 28192237

[B23] Pham J. Su L. D. Hanson K. E. Hogan C. A. (2023). Sequence-based diagnostics and precision medicine in bacterial and viral infections: from bench to bedside. Curr. Opin. Infect. Dis. 36, 228. doi: 10.1097/QCO.0000000000000936 37431553

[B24] Prabhu S. R. (2023). “Infectious and communicable diseases: an overview[M]//prabhu S R ,” in Textbook of general pathology for dental students (Springer Nature Switzerland, Cham), 63–72.

[B25] Qian Y. Wang H. Zhou Y. Zhang H. C. Zhu Y. M. Zhou X.. (2021). Improving pulmonary infection diagnosis with metagenomic next generation sequencing. Front. Cell. Infect. Microbiol. 10, 567615. doi: 10.3389/fcimb.2020.567615 33585263 PMC7874146

[B26] Shen H. Liu T. Shen M. Zhang Y. Chen W. Chen H.. (2023). Utilizing metagenomic next-generation sequencing for diagnosis and lung microbiome probing of pediatric pneumonia through bronchoalveolar lavage fluid in pediatric intensive care unit: results from a large real-world cohort. Front. Cell. Infection Microbiol. 13. doi: 10.3389/fcimb.2023.1200806 PMC10466250 37655299

[B27] Simner P. J. Miller S. Carroll K. C. (2018). Understanding the promises and hurdles of metagenomic next-generation sequencing as a diagnostic tool for infectious diseases. Clin. Infect. Dis. 66, 778–788. doi: 10.1093/cid/cix881 29040428 PMC7108102

[B28] Stratton C. W. Tang Y. (2020). Diagnosing bacteremia in real time using next-generation sequencing–based technology. J. Mol. Diagnostics 22, 301–303. doi: 10.1016/j.jmoldx.2020.01.002 31978560

[B29] Van Seventer J. M. Hochberg N. S. (2017). “Principles of infectious diseases: transmission, diagnosis, prevention, and control[M]//quah S R ,” in International encyclopedia of public health, 2nd ed. (Academic Press, Oxford), 22–39.

[B30] Wang J. Han Y. Feng J. (2019). Metagenomic next-generation sequencing for mixed pulmonary infection diagnosis. BMC Pulm. Med. 19, 252. doi: 10.1186/s12890-019-1022-4 31856779 PMC6921575

[B31] Wei P. Wu L. Li Y. Shi J. Luo Y. Wu W.. (2022). Metagenomic next-generation sequencing for the detection of pathogenic microorganisms in patients with pulmonary infection. Am. J. Trans. Res. 14, 6382–6388. PMC9556471 36247251

[B32] Wu D. Wang W. Xun Q. Wang H. Liu J. Zhong Z.. (2022). Metagenomic next-generation sequencing indicates more precise pathogens in patients with pulmonary infection: A retrospective study. Front. Cell. Infection Microbiol. 12. doi: 10.3389/fcimb.2022.977591 PMC9585196 36275015

[B33] Xiao Y. H. Liu M. F. Wu H. Xu D. R. Zhao R. (2023). Clinical efficacy and diagnostic value of metagenomic next-generation sequencing for pathogen detection in patients with suspected infectious diseases: A retrospective study from a large tertiary hospital. Infect. Drug Resist. 16, 1815–1828. doi: 10.2147/IDR.S401707 37016633 PMC10066896

[B34] Xiao Y. H. Luo Z. X. Wu H. W. Xu D. R. Zhao R. (2024). Metagenomic next-generation sequencing for the identification of infections caused by Gram-negative pathogens and the prediction of antimicrobial resistance. Lab. Med. 55, 71–79. doi: 10.1093/labmed/lmad039 37253164

[B35] Xie Y. Du J. Jin W. Teng X. Cheng R. Huang P.. (2019). Next generation sequencing for diagnosis of severe pneumonia: China, 2010-2018. J. Infect. 78, 158–169. doi: 10.1016/j.jinf.2018.09.004 30237069

[B36] Xie G. Zhao B. Wang X. Bao L. Xu Y. Ren X.. (2021). Exploring the clinical utility of metagenomic next-generation sequencing in the diagnosis of pulmonary infection. Infect. Dis. Ther. 10, 1419–1435. doi: 10.1007/s40121-021-00476-w 34117999 PMC8322361

[B37] Zhao Z. Chen X. Wang Y. Feng J. (2023). Comparison of quality/quantity mNGS and usual mNGS for pathogen detection in suspected pulmonary infections. Front. Cell. Infection Microbiol. 13. doi: 10.3389/fcimb.2023.1184245 PMC10425550 37588054

[B38] Zheng Y. Qiu X. Wang T. Zhang J. Zhang J. (2021). The diagnostic value of metagenomic next–generation sequencing in lower respiratory tract infection. Front. Cell. Infection Microbiol. 11. doi: 10.3389/fcimb.2021.694756 PMC8458627 34568089

[B39] Zhou H. Larkin P. M. K. Zhao D. Ma Q. Yao Y. Wu X.. (2021). Clinical impact of metagenomic next-generation sequencing of bronchoalveolar lavage in the diagnosis and management of pneumonia. J. Mol. Diagnostics 23, 1259–1268. doi: 10.1016/j.jmoldx.2021.06.007 34197923

